# Bamboo charcoal affects soil properties and bacterial community in tea plantations

**DOI:** 10.1515/biol-2022-0681

**Published:** 2023-08-08

**Authors:** Si-Hai Zhang, Yue Wang, Jin-Jie Hu, Wei-Jia Chen, Jia-Le Wu, Rachel Wan Xin Seah, Yang-Chun Zhu, Zhi-Ping Guo, Jie Chen

**Affiliations:** College of Liangshan, Lishui University, Lishui 323000, China; College of Horticulture Science, Zhejiang A&F University, Hangzhou 311300, China; College of Ecology, Lishui University, No. 1 Xueyuan Road, Lishui 323000, China; Department of Biological Science, National University of Singapore, Singapore 117558, Singapore

**Keywords:** soil physicochemistry, soil enzyme, Illumina NovaSeq, soil microbiota

## Abstract

Bamboo charcoal, a type of manufactured biochar, is produced by pyrolyzing bamboo residue under anoxic conditions. Its beneficial properties in absorption, catalyst support, and agricultural function have attracted significant attention; however, relatively few studies have examined its effects on the soil microbiota. In this study, we analyzed the effects of bamboo charcoal on soil physicochemical properties, enzymes, and microbial community structure in tea plantations and investigated the optimal amount of bamboo charcoal to be added to organic fertilizer. The results show that bamboo charcoal can further increase soil available nitrogen, total and available phosphorus and potassium, organic carbon content, pH, and urease activity. However, only the combined use of bamboo charcoal and organic fertilizer significantly increased total nitrogen, sucrase, and β-glucosidase activities in the soil. Bamboo charcoal also significantly increased the Chao1 and Shannon indices of microbiota diversity in a concentration-dependent manner. The structure of the bacterial community changed significantly after the bamboo charcoal addition, with *Proteobacteria*, *Actinobacteria*, and *Firmicutes* increasing and *Acidobacteria* decreasing. This study provides fundamental insights into the suitability of bamboo charcoal application for the ecological remediation of diseased soils.

## Graphical abstract

**Figure:**
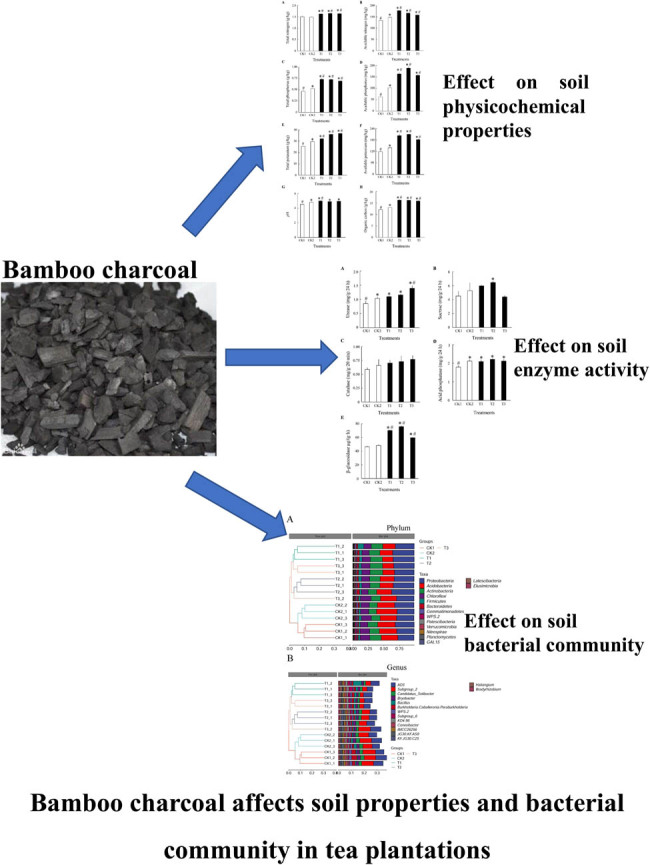

## 1 Introduction

Soil is essential for tea tree survival, and its growth, characteristics, and nutrient richness directly affect tea tree growth and leaf quality [1]. Tea trees thrive in acidic soils; however, only a suitable soil pH supports their root system survival [2]. In studies on continuous crop barriers in tea trees, it was found that soil nutrient imbalance, soil acidification, self-toxic effects, microbial community structure imbalance, and inter-root microecology pose continuous difficulties in tea plant cropping [3,4,5], which can be attributed to the economic interests of tea farmers and result in massive chemical fertilizer application [3,4].

Biochar, a class of insoluble organic matter with highly aromatized structures, is produced by high-temperature pyrolysis of plant or animal biomass materials under oxygen-limited conditions [6]. Biochar has a high carbon-to-nitrogen ratio, rich pore structure, large specific surface area, stable physicochemical properties, high soil cation exchange capacity, weak alkalinity, and low density [7]. It can be employed to improve soil pH and organic carbon content, increase soil fertility and water retention, and as a soil conditioner to promote crop growth and development [8,9,10]. Application of biochar to Karst lime soil increased soil pH, moisture, and porosity, and the soil’s phosphatase activity was enhanced due to the size of the biochar particles [11]. A study on the effect of biochar in combination with organic-inorganic fertilizer in pomelo orchards found that after 3 years, this combination had improved soil nutrients (pH, alkali hydrolysable nitrogen, and available phosphorus (AP), potassium, and magnesium), enzymatic activities (urease, dehydrogenase, invertase, and nitrate reductase activities), and bacterial abundance [12]. In addition, biochar can improve the soil microhabitat, which benefits the soil microbial habitat and activity and protects beneficial soil microorganisms [13,14].

Bamboo charcoal, a type of manufactured biochar, is produced by pyrolyzing bamboo residue under anoxic conditions. It has attracted significant attention owing to its beneficial properties in absorption, catalyst support, and agricultural function [15,16,17,18]. Furthermore, bamboo charcoal has an excellent absorption ability for most pollutants [19]. For example, it can reduce soil fluoride availability and subsequent fluoride uptake by tea plants [20]. In addition, it promotes tea tree growth, as one study found a 20–40% growth increase over the control group [21]. This could be attributed to its manifold benefits such as adjusting soil pH, improving the soil structure, facilitating root growth of plants, enhancing nutrient absorption, and reduces the rate of nutrient loss in the soil [22].

Over an extended period of continuous crop use, the structure of the soil microbial community between the roots of tea trees changes dramatically. This includes a decrease in the number of soil microorganisms, a decrease in community diversity, an increase in microbiota with low metabolic capacity or better suitability to poor conditions, and an overall decrease in the quality of the soil environment between the roots [23,24]. The imbalance in soil microbial community structure is therefore one of the most important causes of crop barriers in tea plants. Bamboo charcoal is increasingly being used to address this problem because of its multiple excellent properties [13,25]. However, there are currently limited studies on the effects of bamboo charcoal on the microbial community structure of tea trees. In this study, the effects of bamboo charcoal on the soil microbial community were analyzed by 16S rDNA sequencing. The study will help to elucidate the potential mechanisms by which bamboo charcoal affects the structure of soil microbial communities.

## 2 Materials and methods

### 2.1 Soil

The soil used was sourced from a farm in Suichang County, Zhejiang Province, China, located at 28°13′N–28°49′N, 118°41′E–119°30′E. The farm serves as a tea plantation, and the area has a subtropical monsoon climate with an annual average temperature of 16.8℃ with extreme maximum and minimum temperatures of 40.1 and −9.9℃, respectively, and annual average sunshine of 1346.5–1847.8 h. Given that tea trees are perennial plants, soil acidification becomes serious after 10 years of continuous planting and tea quality declines, posing significant continuous cropping obstacles. The soil characteristics were as follows: organic carbon, 20.92 g/kg; available nitrogen (AN), 1357.65 mg/kg; AP, 65.60 mg/kg; available potassium, 119.84 mg/kg; and pH, 4.52.

### 2.2 Plot selection and soil collection

Fifteen plots of land of 8 m × 8 m were set up on the farm. The five treatments include CK1, control treatment without fertilizer; CK2, control treatment with organic fertilizer 7,500 kg/hm^2^; T1 treatment, organic fertilizer 7,500 kg/hm^2^ + bamboo charcoal 1,125 kg/hm^2^; T2 treatment, organic fertilizer 7,500 kg/hm^2^ + bamboo charcoal 2,250 kg/hm^2^; T3 treatment, organic fertilizer 7,500 kg/hm^2^ + bamboo charcoal 3,375 kg/hm^2^. Three replicates of each treatment were used in a randomized block treatment design. The bamboo charcoal used was bamboo straw charred at 800℃ and sieved using 80 mesh, and the organic fertilizer was a common market organic fertilizer. Treatment was applied on November 9, 2017; November, 2018; and November, 2019, using open furrows for even application followed by mulching. Soil samples covering a depth of 0–20 cm were obtained from each plot using an S-shape on June 10, 2020.

### 2.3 Soil chemical analysis

Soil pH was determined via soil liquid extraction (water/soil = 2.5/1, v/w) using a pH meter [26]. Soil total nitrogen (TN) was determined using the Kjeldahl distillation method [27]. Soil total potassium (TK) was determined with a flame photometer (FP6410, China) [26]. Soil total phosphorus (TP) was determined by NaOH fusion, Mo–Sb colorimetry, and ultraviolet spectrophotometry (UV2600, Shimadzu, Japan) [28]. AN was measured by alkaline hydrolysis diffusion [29], and AP was analyzed by sodium bicarbonate extraction molybdenum antimony resistance colorimetry [29]. Available potassium (AK) was measured by NH_4_CH_3_CO_2_ extraction and atomic absorption spectrometry [26]. The content of soil organic matter (SOM) was determined by the hydrothermal potassium dichromate oxidation colorimetric method [29].

### 2.4 Soil enzyme activity analysis

The soil urease activity was determined by the sodium phenol-sodium hypochlorite colorimetric method using an MD SpectraMax 190 Fluorescence Microplate Reader at 578 nm [30]. Soil acid phosphatase activity was determined by the colorimetric method using sodium benzene phosphate at 570 nm [31]. Peroxidase activity was determined by the potassium permanganate titration method [32]. Sucrase was determined by a colorimetric method using 3,5-dinitrosalicylic acid at 508 nm [33,34]. β-Glucosidase activity was determined by colorimetric method using *p*-nitrophenol at 400 nm [35].

### 2.5 DNA extraction

Three fresh soil samples from each of the five treatments (CK1, CK2, T1, T2, and T3) were collected for genomic DNA (gDNA) extraction. Total gDNA was extracted using the OMEGA Soil DNA Kit (Omega Bio-Tek, Norcross, GA, USA). A NanoDrop NC2000 spectrophotometer (Thermo Fisher Scientific, Waltham, MA, USA) was used and agarose gel electrophoresis was performed to ensure the quantity and quality of the gDNA.

### 2.6 16S rRNA gene amplicon sequencing

16S rRNA gene sequencing was performed as in a previous study [36]. PCR amplification of bacterial 16S rRNA gene V3-V4 was performed using forward primer 338F (5′-ACTCCTACGGGAGGCAGCA-3′) and reverse primer 806R (5′-GGACTACHVGGGTWTCTAAT-3′). For multiplex sequencing, unique barcodes around 7 base pairs in length were incorporated into the primers. The PCR components contained 5 μL of buffer (5×), 0.25 μL of Fast pfu DNA Polymerase (5 U/μL), 2 μL (2.5 mM) of deoxynucleotide triphosphates (dNTPs), 1 μL (10 μM) of each forward and reverse primer, 1 μL of DNA template, and 14.75 μL of ddH_2_O. Thermal cycling included initial denaturation at 98°C for 5 min, followed by 25 cycles of denaturation at 98°C for 30 s, annealing at 53°C for 30 s, and extension at 72°C for 45 s, with a final extension of 5 min at 72°C. The amplicons were purified using Vazyme VAHTSTM DNA Clean Beads (Vazyme, Nanjing, China) and quantified using the Quant-iT PicoGreen dsDNA Assay Kit (Invitrogen, Carlsbad, CA, USA). The amplicons were pooled and then sequenced on the Illumina NovaSeq platform with NovaSeq 6000 SP Reagent Kit (500 cycles) at Shanghai Personal Biotechnology Co., Ltd (Shanghai, China).

### 2.7 Sequence analysis

Microbiome bioinformatics was performed using QIIME2 2019.4, with slight modifications. *Demux*, *cutadapt,* and *DADA2* were used for quality control and preparation for further analysis. A phylogenetic tree was constructed using *fasttree2* by aligning non-singleton amplicon sequence variants (ASVs) with MAFFT. Alpha-diversity metrics and beta-diversity metrics were calculated with *diversity.* ASVs were assigned taxonomy using the classify-sklearn naïve Bayes taxonomy classifier in the *feature-classifier* plugin against the SILVA Release 132 Database.

### 2.8 Statistical analysis

One-way analysis of variance (ANOVA) was conducted in SPSS version 13.0 (SPSS Inc., Chicago, IL, USA). All data are expressed as mean ± standard error of the mean (SEM). Results were considered to be statistically significant at *P* < 0.05.

## 3 Results

### 3.1 Effect of bamboo charcoal on soil physicochemical properties

**Figure 1:**
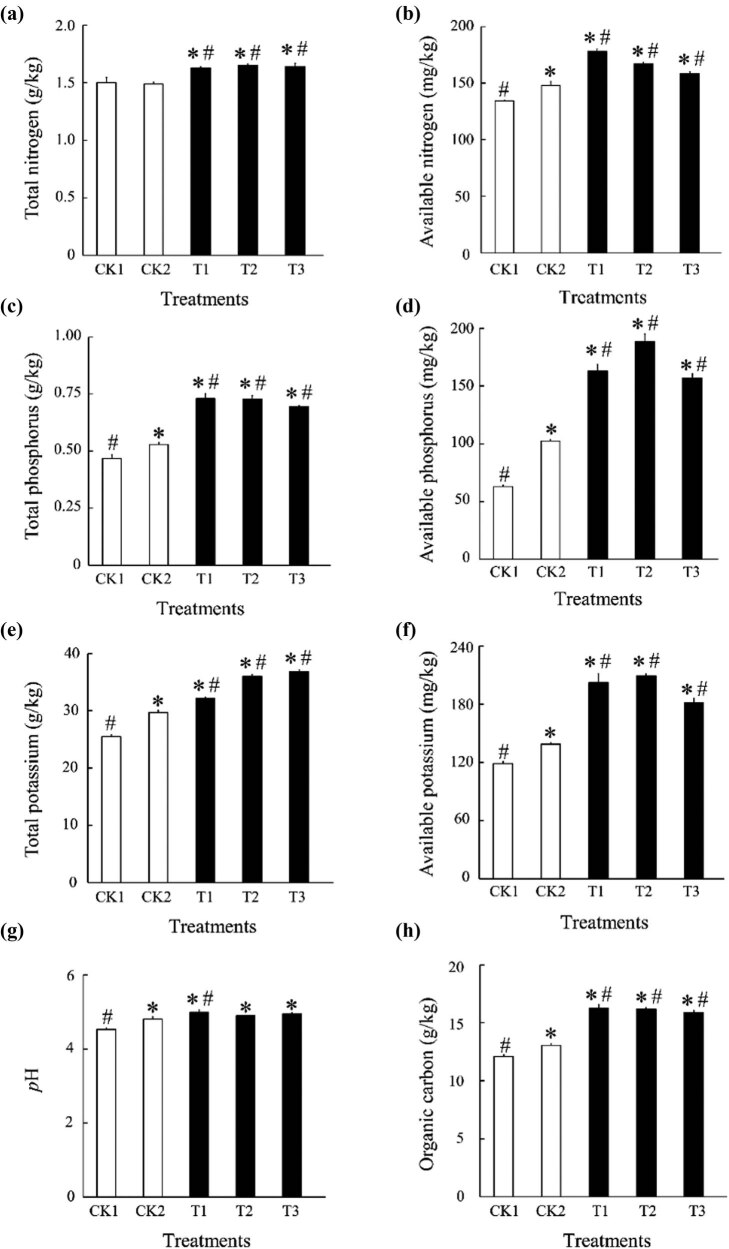
Effect of bamboo charcoal on soil physicochemical properties. (a) total nitrogen; (b) available nitrogen; (c) total phosphorus; (d) available phosphorus; (e) total potassium; (f) available potassium; (g) *p*H; (h) organic carbon; CK1, without fertilizer; CK2, organic fertilizer 7,500 kg/hm^2^; T1 treatment, organic fertilizer 7,500 kg/hm^2^ + bamboo charcoal 1,125 kg/hm^2^; T2 treatment, organic fertilizer 7,500 kg/hm^2^ + bamboo charcoal 2,250 kg/hm^2^; T3 treatment, organic fertilizer 7,500 kg/hm^2^ + bamboo charcoal 3,375 kg/hm^2^. Data are expressed as the mean ± SEM of the results from three samples. The values calculated using ANOVA were denoted by * and #, and are significantly different from CK1 and CK2, respectively (*P* < 0.05).

Soil TN, AN, TP, AP, TK, AK, and organic carbon differed significantly from that of the control groups CK1 and CK2 with increased bamboo charcoal (Figure 1). TN and TK increased with elevated bamboo charcoal concentration, whereas AN, TP, and organic carbon decreased. AP and AK increased and then decreased with elevated bamboo charcoal concentration. For pH, only the T1 treatment was significantly different from CK1 and CK2, whereas the T2 and T3 treatments were only significantly different from CK1.

### 3.2 Effect of bamboo charcoal on soil enzyme activity

**Figure 2:**
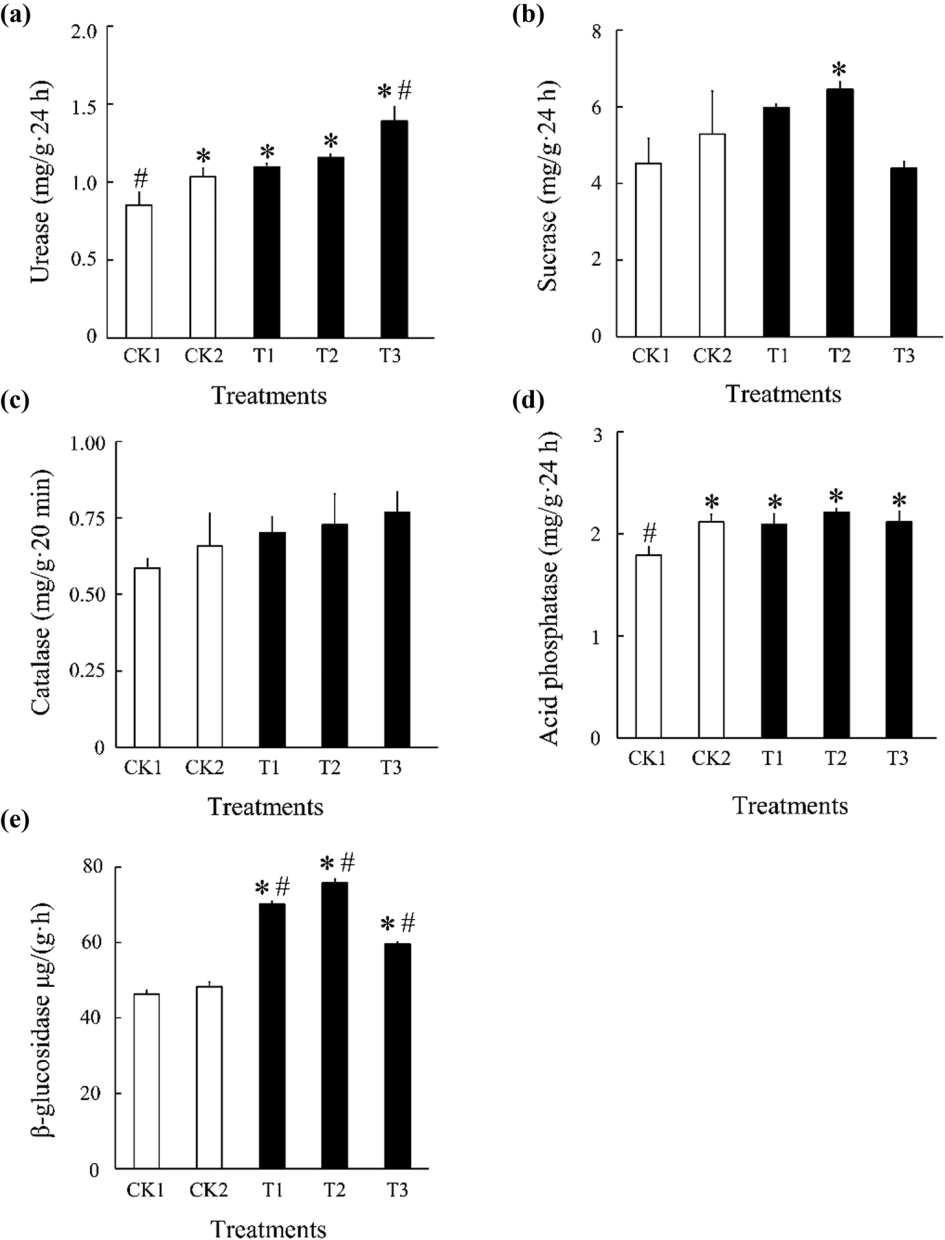
Effect of bamboo charcoal on soil enzyme activity. (a) urease; (b) sucrase; (c) catalase; (d) acid phosphatase; (e) β-glucosidase; CK1, without fertilizer; CK2, organic fertilizer 7,500 kg/hm^2^; T1 treatment, organic fertilizer 7,500 kg/hm^2^ + bamboo charcoal 1,125 kg/hm^2^; T2 treatment, organic fertilizer 7,500 kg/hm^2^ + bamboo charcoal 2,250 kg/hm^2^; T3 treatment, organic fertilizer 7,500 kg/hm^2^ + bamboo charcoal 3,375 kg/hm^2^. The three samples are represented as the mean ± SEM of the results. The values denoted by * and # are significantly different from CK1 and CK2, respectively, when compared using one-way ANOVA (*P* < 0.05).

With increased bamboo charcoal concentration, the urease activity gradually increased; however, only the T3 treatment was significantly different from CK1 and CK2, and T1 and T2 were only significantly different from CK1. Sucrase enzyme activity increased and then decreased, and only the T2 treatment was significantly different from CK1. Catalase enzyme activity gradually increased, but not significantly, with CK1 and CK2. Acid phosphatase enzyme activity increased and then decreased but was only significantly different in CK1. β-Glucosidase enzyme activity increased significantly, with an initial increase followed by a decrease (Figure 2).

### 3.3 Sequencing results

**Figure 3:**
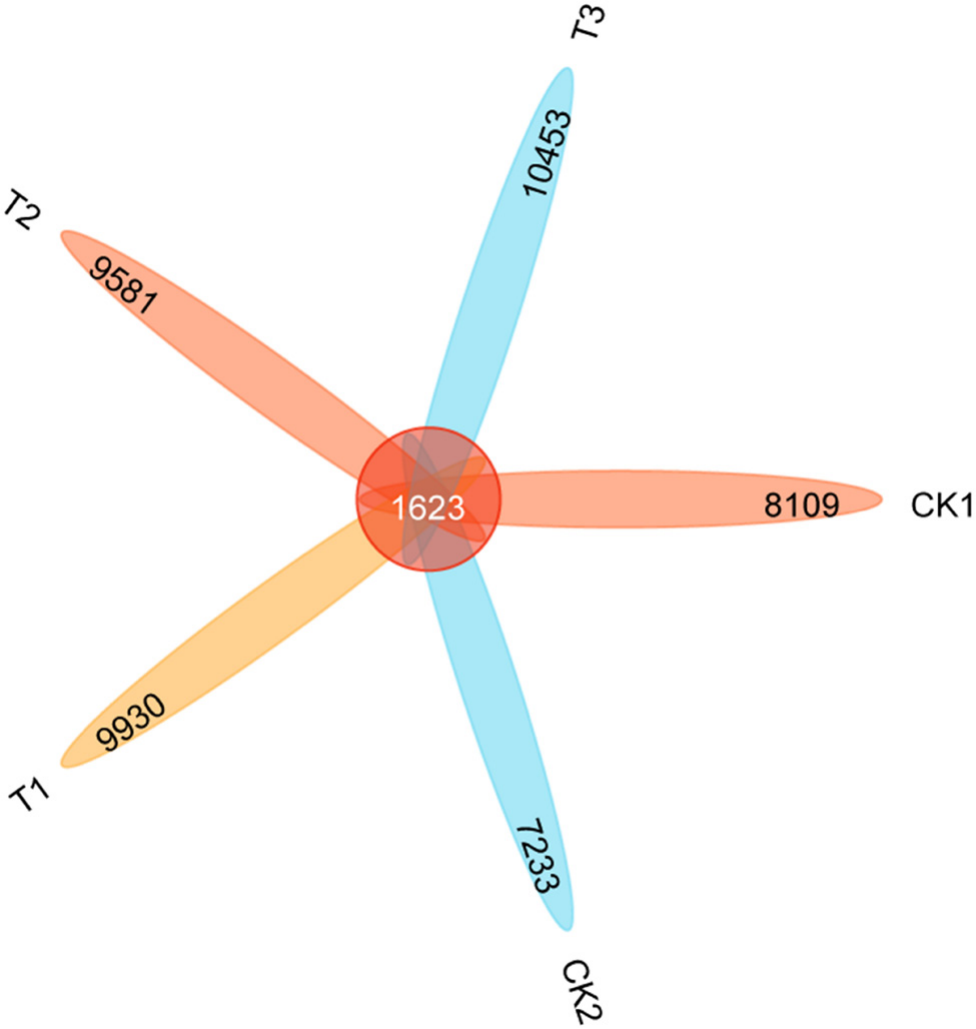
The number of OTUs in the bacterial community of each treatment. CK1, without fertilizer; CK2, organic fertilizer 7,500 kg/hm^2^; T1 treatment, organic fertilizer 7,500 kg/hm^2^ + bamboo charcoal 1,125 kg/hm^2^; T2 treatment, organic fertilizer 7,500 kg/hm^2^ + bamboo charcoal 2,250 kg/hm^2^; T3 treatment, organic fertilizer 7,500 kg/hm^2^ + bamboo charcoal 3,375 kg/hm^2^.

The data obtained from 16 S rRNA sequencing were uploaded to the NCBI SRA database, and the acceptance number PRJNA970211 was obtained. The total number of operational taxonomic units (OTUs) detected from samples in this study was 45,306, the total number of OUTs common to all samples was 1,623, and the total number of OUTs specific to each treatment was 6,486 (CK1), 5,610 (CK2), 8,307 (T1), 7,958 (T2) and 8,830 (T3), respectively. Overall, the application of organic fertilizer reduced the total number of soil OTUs, while the addition of bamboo charcoal increased it (Figure 3).

### 3.4 Soil bacterial community: α diversity

With increased bamboo charcoal concentration, the Chao1 and observed species index of T1, T2, and T3 significantly increased compared to the control groups CK1 and CK2, indicating an increase in soil microbiota richness. The Shannon index increased, especially T3, which was significantly higher than CK2, indicating an increase in soil microbiota diversity and uniformity. However, Simpson and Good’s coverage index did not change significantly (Table 1).

**Table 1:** Effect of bamboo charcoal on the α diversity of soil bacterial communities

Treatment	Chao1	Observed species	Shannon	Simpson	Good’s coverage
CK1	6,530 ± 172^b^	6,231 ± 223^b^	11.12 ± 0.06^ab^	1.00 ± 0.00^a^	1.00 ± 0.00^a^
CK2	6,368 ± 235^b^	5,997 ± 231^b^	11.07 ± 0.13^b^	1.00 ± 0.00^a^	1.00 ± 0.00^a^
T1	7,391 ± 315^a^	7,083 ± 321^a^	11.44 ± 0.15^ab^	1.00 ± 0.00^a^	1.00 ± 0.00^a^
T2	7,397 ± 433^a^	7,074 ± 359^a^	11.45 ± 0.07^ab^	1.00 ± 0.00^a^	1.00 ± 0.00^a^
T3	7,761 ± 392^a^	7,442 ± 421^a^	11.52 ± 0.21^a^	1.00 ± 0.00^a^	1.00 ± 0.00^a^

CK1, without fertilizer; CK2, organic fertilizer 7,500 kg/hm^2^; T1 treatment, organic fertilizer 7,500 kg/hm^2^ + bamboo charcoal 1,125 kg/hm^2^; T2 treatment, organic fertilizer 7,500 kg/hm^2^ + bamboo charcoal 2,250 kg/hm^2^; T3 treatment, organic fertilizer 7,500 kg/hm^2^ + bamboo charcoal 3,375 kg/hm^2^. Data are expressed as the mean ± SEM. Values denoted by different letters are significantly different between groups when compared using one-way ANOVA (*P* < 0.05).

### 3.5 Soil bacterial community: β diversity

**Figure 4:**
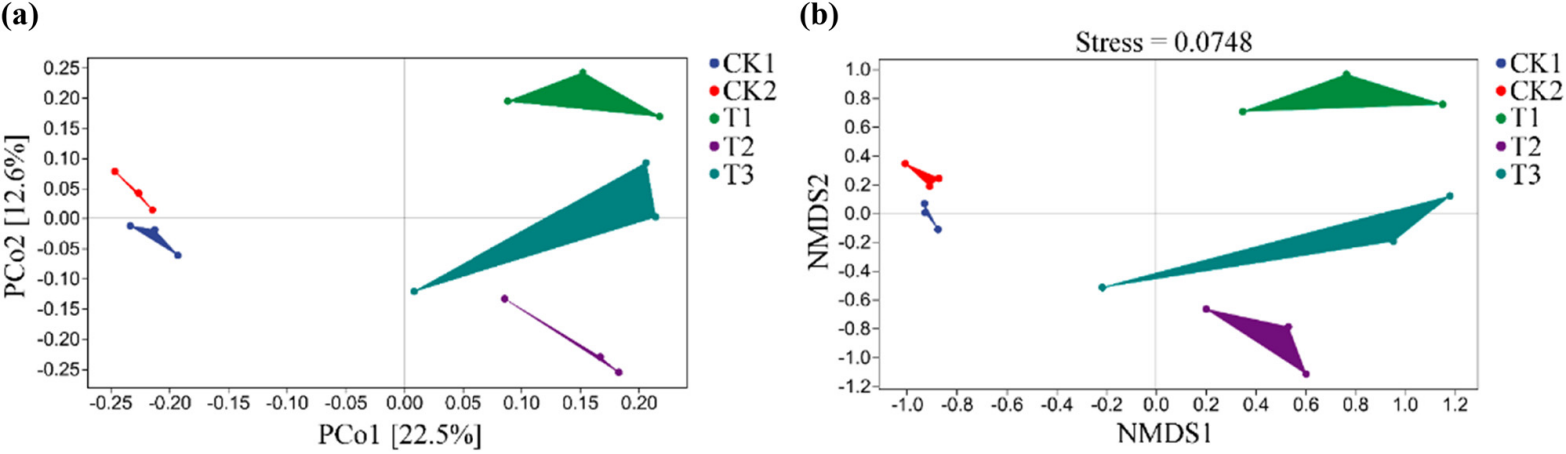
The effect of bamboo charcoal on the β diversity of soil bacterial communities. The control and bamboo charcoal-treated samples were visualized by PCoA (a) and NMDS (b) analysis. CK1, without fertilizer; CK2, organic fertilizer 7,500 kg/hm^2^; T1 treatment, organic fertilizer 7,500 kg/hm^2^ + bamboo charcoal 1,125 kg/hm^2^; T2 treatment, organic fertilizer 7,500 kg/hm^2^ + bamboo charcoal 2,250 kg/hm^2^; T3 treatment, organic fertilizer 7,500 kg/hm^2^ + bamboo charcoal 3,375 kg/hm^2^.

The principal coordinate analysis (PCoA) revealed that the control groups CK1 and CK2 were similar regarding bacterial community structure, whereas they differed from that of the bamboo charcoal treatment groups (T1, T2, and T3 [Figure 4a]). The nonmetric multidimensional scaling (NMDS) analysis results corroborated those of the PCoA (Figure 4b).

### 3.6 Bacterial identification and species distribution

**Figure 5:**
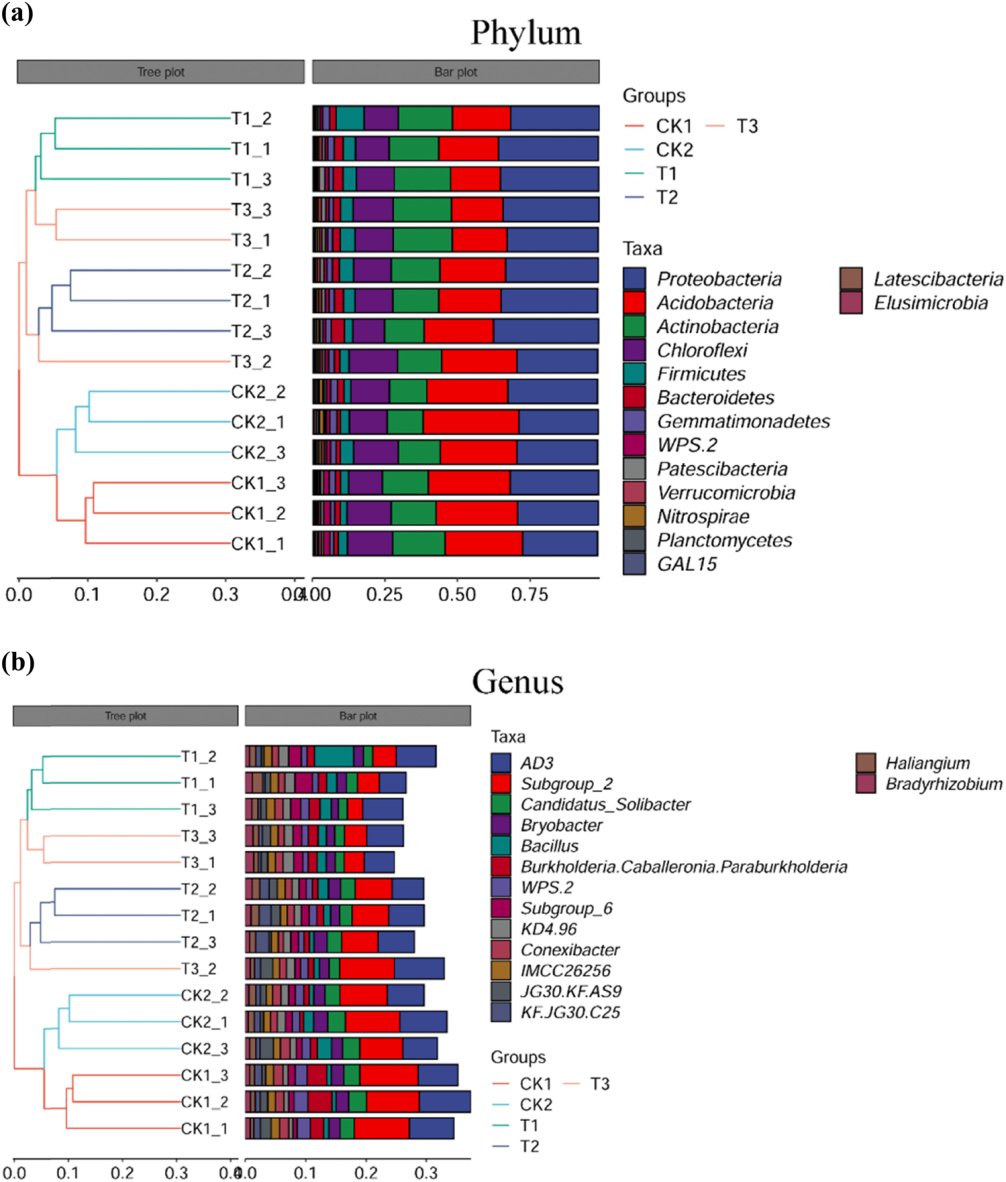
Bacterial composition of the control and bamboo charcoal treatment groups at the phylum and genus levels. Only the top 15 most abundant bacterial phylum (a)/genera (b) (based on relative abundance) are listed. CK1, without fertilizer; CK2, organic fertilizer 7,500 kg/hm^2^; T1 treatment, organic fertilizer 7,500 kg/hm^2^ + bamboo charcoal 1,125 kg/hm^2^; T2 treatment, organic fertilizer 7,500 kg/hm^2^ + bamboo charcoal 2,250 kg/hm^2^; T3 treatment, organic fertilizer 7,500 kg/hm^2^ + bamboo charcoal 3,375 kg/hm^2^.

The phyla *Proteobacteria*, *Acidobacteria*, *Actinobacteria*, *Chloroflexi*, and *Firmicutes* dominated the soil microbiota in the control and bamboo charcoal treatment groups, but community composition differed between control and treatment groups (Figure 5a). *AD3*, *Subgroup_2*, *Candidatus_Solbacter*, *Bryobacter*, *Bacillus*, and *Burkholderia–Caballeronia–Paraburkholderia* dominated the soil microbiota in the control and bamboo charcoal treatment groups at the genus level, but community composition differed significantly between control and treatment groups (Figure 5b).

### 3.7 Identification of potential soil microbial biomarker

**Figure 6:**
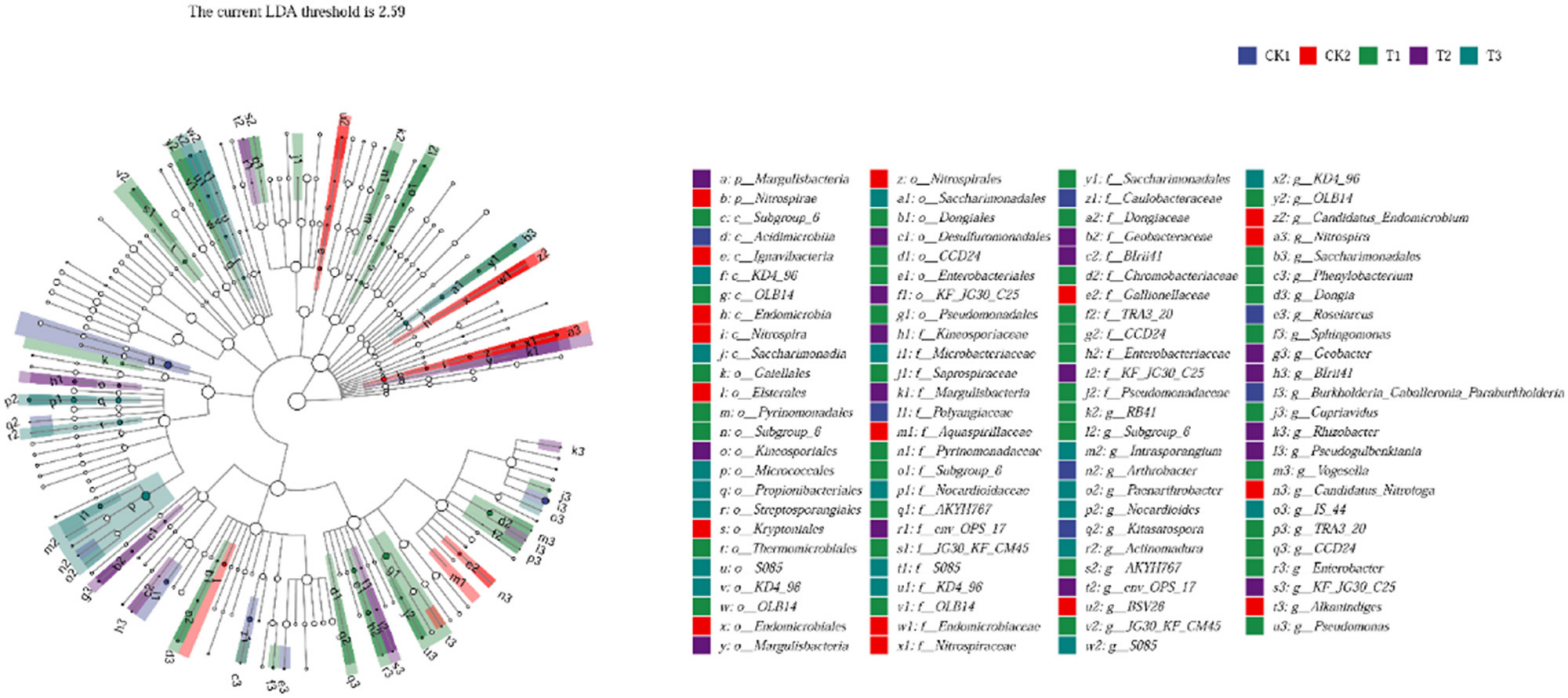
Visualization of the differential abundance of soil bacterial taxa between the control and bamboo charcoal treatment groups using LEfSe analysis. The bacterial phylum is represented by center dots while the outer circle of dots represents the OTUs at the species level. The color and labels are linked to the respective of the listed abundant bacterial taxa. CK1, without fertilizer; CK2, organic fertilizer 7,500 kg/hm^2^; T1 treatment, organic fertilizer 7,500 kg/hm^2^ + bamboo charcoal 1,125 kg/hm^2^; T2 treatment, organic fertilizer 7,500 kg/hm^2^ + bamboo charcoal 2,250 kg/hm^2^; T3 treatment, organic fertilizer 7,500 kg/hm^2^ + bamboo charcoal 3,375 kg/hm^2^.

Linear discriminant analysis effect size (LEfSe) was used to characterize the microbial communities. There was a significant difference in abundance between the control and bamboo charcoal treatment groups. The relative abundance of *Burkholderia–Caballeronia–Paraburkholderia*, *Arthrobacter*, *Roseiarcus*, and *Kitasatospora* were significantly higher in the CK1 group, whereas that of *Nitrospira*, *Alkanindiges*, *Candidatus_Nitrotoga*, and *Candidatus_Endomicrobium* were significantly higher in the CK2 group. However, the biomarker bacteria changed slightly between the treatment groups as bamboo charcoal concentration increased. *Saccharimonadales*, *Cupriavidus*, *Sphingomonas*, and *Phenylobacterium* dominated the T1 treatment group; *Geobacter*, *Pseudogulbenkiania*, and *Rhizobacter* dominated the T2 treatment group; and *Nocardioides*, *Paenarthrobacter*, and *Actinomadura* dominated the T3 treatment group (Figure 6).

## 4 Discussion

Bamboo charcoal has attracted significant attention owing to its beneficial properties in absorption, catalyst support, and agricultural function [15,16,17,18]. However, relatively few studies have examined its effects on soil bacterial community structure. In this study, bamboo charcoal significantly increased the soil pH, which can be attributed to its alkalinity and ability to increase the pH of acidic and neutral soils [37]. Furthermore, the study suggests that bamboo charcoal increased the organic carbon content of the soil. The potential carbon sequestration ability of biochar was investigated by Hua et al. [38], who showed that biochar can effectively reduce CO_2_ emissions and increase organic matter content. In soil with lower organic content, increasing biochar enhances soil efficacy level at a much higher rate than soil with higher organic content [38]. Biochar also increases nitrogen retention in soil by reducing gaseous loss to the environment and maintains phosphorous availability for plant growth by decreasing the rate of leaching. However, for K and other nutrients, there appears to be an inconsistency in the effects of biochar on the soil with a mixed array of positive and negative effects [39]. In the present study, bamboo charcoal significantly increased the total amount and effectiveness of soil N, P, and K.

Bamboo charcoal treatment significantly increased urease, sucrase, acid phosphatase, and β-glucosidase activities. β-Glucosidase plays a key role in cellulose degradation, and its degradation products are the main energy sources for soil microorganisms [40]. In the present study, β-glucosidase activity was higher in the bamboo charcoal treatment group, which might be attributed to the ability of the bamboo charcoal to improve plant growth microenvironment, inducing better plant growth and increased belowground root biomass, aboveground apoplankton, and SOM accumulation, consequently providing more available substrates to increase β-glucosidase activity. It is known that acid phosphatase activity directly affects the catabolic transformation and bioefficacy of organic P in soil [41]. In this study, acid phosphatase activity was higher in the bamboo charcoal treatment, which might be attributed to the better growth and higher biomass of the bamboo charcoal-treated plants, which could have stimulated the microorganisms to secrete more acidic P. In contrast, urease activity depends on organic matter and is deeply involved in the conversion and decomposition of organic matter [42]. As the study revealed that bamboo charcoal increased the organic matter content, and the urease activity of bamboo charcoal treatment groups was consequently higher, as there was more substrate available for urease to work on. The presence of sucrase in soil aids in carbohydrate conversion, and it is one of a key indicator in soil fertility [43]. In this study, soil sucrase activity was enhanced with increased bamboo charcoal application, consistent with previous studies [44].

The results indicate that soil microbiota diversity and richness significantly increased with elevated bamboo charcoal concentration. Zhang et al. [45] observed the effects of biochar addition in citrus production on microbial communities, and the experimental results showed that biochar significantly affected soil microbiota richness, evenness, and diversity in citrus orchards. The results are also consistent with a previous study in which soil microbiota was largely governed by organic matter utilization [46]. The most dominant soil microbiota phyla in the present study were similar to those in previous studies of bacterial communities in different types of soils [47,48]. However, differences were observed in the composition of soil bacterial communities in this study. Changes in the relative bacterial abundance mainly occurred in the *Proteobacteria*, *Acidobacteria*, *Actinobacteria*, *Chloroflexi*, and *Firmicutes* phyla. Bamboo charcoal addition increased the relative abundances of *Proteobacteria*, *Actinobacteria*, and *Firmicutes*, whereas that of *Acidobacteria* decreased. Studies have reported that *Proteobacteria*, *Actinobacteria*, and *Firmicutes* were found to easily dominate an environment rich in active organic carbon [49,50]. Since bamboo charcoal application increased soil active organic carbon availability, this resulted in an increase in the relative abundance of *Proteobacteria*, *Actinobacteria*, and *Firmicutes*. As *Acidobacteria* proliferate in anaerobic conditions, the presence of bamboo charcoal can increase soil permeability to air, and potentially cause a reduction in *Acidobacteria* levels [51]. These changes could account for the differences in the soil microbiota structure between the control and bamboo charcoal treatment groups, which were also reflected in the different microbial biomarkers of each treatment group. Li et al. [23,24] found that the richness, evenness, and diversity indices of soil microbiota in the inter-rhizosphere of *Camellia sinensis* decreased significantly with increasing planting years, and that some genera of bacteria beneficial to plant growth (e.g., *Pseudomonas*, *Flavobacterium*, and *Rhizobium*) decreased sharply, while the number of certain pathogenic fungi (e.g., *Streptomyces*) increased. However, research on the inter-root microorganisms of tea plants is currently still limited to phenomenological descriptions. The key specific microorganisms that play an important role in the formation of tea crop barriers have not yet been elucidated. Further studies using different analysis methods should be carried out to extend the findings of the 16 S rDNA sequencing study.

## 5 Practical implications of this study

Since this study used 16S rRNA sequencing rather than metagenomics sequencing to study the effects of bamboo charcoal on soil microbiota, it can only address the domains of soil microbiota composition and diversity, but not those of microbiota genes and functions. Future studies will employ metagenomics sequencing to clarify the effects of bamboo charcoal on soil community genes and functions, and further elucidate the deeper mechanisms of the effects recorded in the present study. This will also facilitate the application of bamboo charcoal in solving crop barriers in tea plants.

## 6 Conclusions

This study has shown that bamboo charcoal can further increase soil physicochemical properties and enzyme activities in tea plantations in the presence of organic fertilizer application and also clarified the effect of bamboo charcoal on soil microbiota composition and abundance. The results provide fundamental insights into the suitability of bamboo charcoal application for the ecological remediation of diseased soils in tea plantations.
